# Supplementary material for: All-gas-phase synthesis of UiO-66 through modulated atomic layer deposition
Source: Nat Commun. 2016 Nov 23;7:13578. doi: 10.1038/ncomms13578 (PMC5123030; doi:10.1038/ncomms13578)
Supplement: Supplementary Information — Supplementary Figures 1-3. [file ncomms13578-s1.pdf]

Supplementary figures

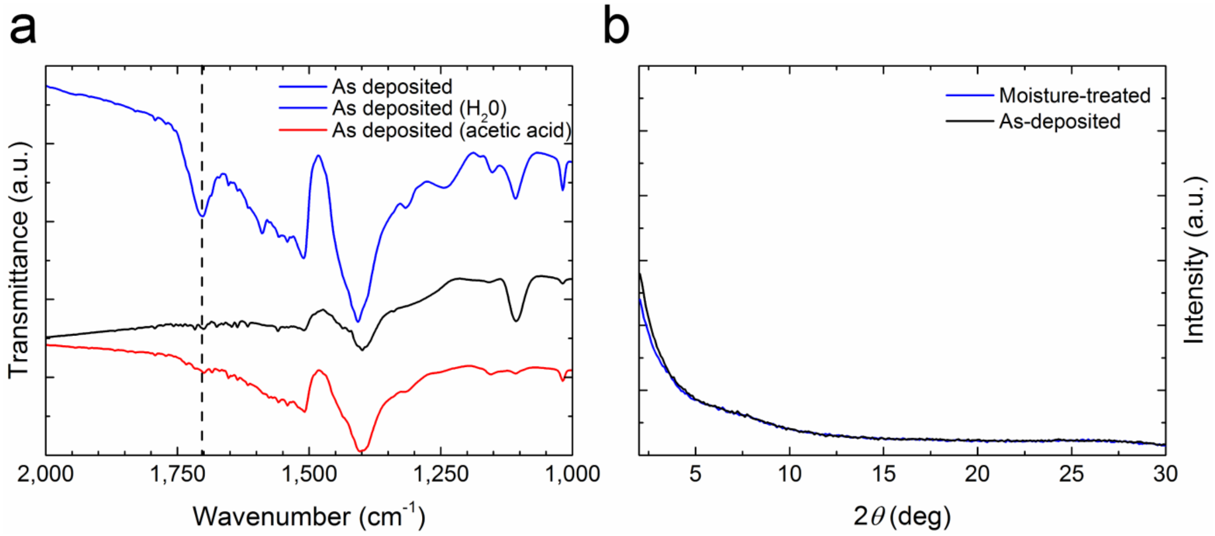

Supplementary Figure 1 | Characterisations of films deposited with H_2O pulsing. a, Fourier transform infrared (FTIR) spectra of the film deposited with H_2O pulses in addition to the two precursors $ZrCl_4$ and 1,4-benzene dicarboxylic acid (1,4-BDC) (black); and an unmodulated sample (blue) and an acetic acid modulated samples (red) for comparison. The peak that indicates a monodentate coordination of 1,4-BDC, which is seen in the unmodulated sample (dashed line), is removed when water or acetic acid is pulsed in addition to the two precursors. **b**, a GIXRD pattern for a film deposited with H_2O pulsing as deposited (black), and a GIXRD pattern for the same film after exposure to a relative humidity of 70-75 % for 24h (blue). No peaks corresponding to crystalline 1,4-BDC are seen on the sample that has been exposed to moisture, indicating that the excess 1,4-BDC is removed similar to the effect seen with acetic acid in Figure 5c.

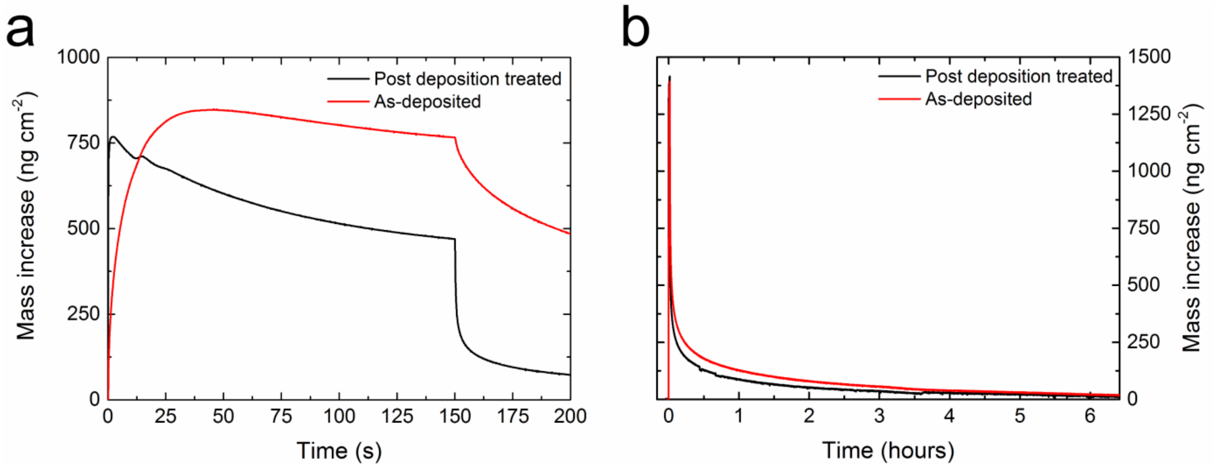

Supplementary Figure 2 | Saturated porosity test. a, porosity test performed by monitoring the water uptake by QCM-crystals during a 150 second water pulse. The test was performed in the ALD reactor at room temperature with a base pressure of 5 mbar N_2. Red is the as-deposited sample (ca. 230 nm) and black is the post deposition treated sample (ca. 500 nm). Both samples saturate with water. The kinetics of the saturation are not investigated further due to too many unknown factors, such as a drop in the partial pressure of water over time during the pulse and a likely difference in pore size in the two films. **b**, a similar porosity test performed with the same samples and a pulse length of 1 minute followed by a long purge. All of the adsorbed mass is lost during the purge. The relative humidity during this pulse was approximately 11% compared to approximately 7% for all the other porosity tests due to an increased water dosage during the pulse.

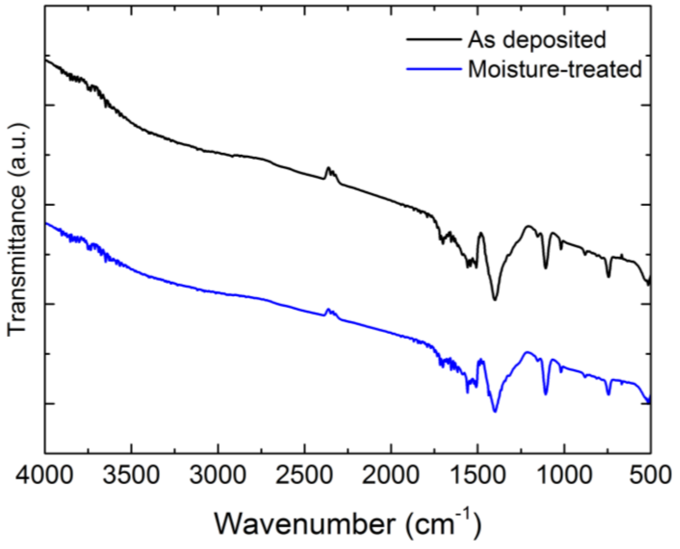

Supplementary Figure 3 | FTIR of moisture treated sample. FTIR spectra of a film deposited with acetic acid modulation, before (black) and after (blue) exposure to a relative humidity of 70-75 % for 24h. No changes occur in the FTIR spectrum upon exposure to moisture. These samples are similar to the ones used for the porosity tests.
